# Dietary patterns, metabolic pathways and metainflammation in hidradenitis suppurativa: a systematic review

**DOI:** 10.3389/fimmu.2026.1830618

**Published:** 2026-05-18

**Authors:** Ezgi Celik, Falk G. Bechara, Eggert Stockfleth, Lennart Ocker-Serger, Nessr Abu Rached

**Affiliations:** 1 International Centre for Hidradenitis Suppurativa/Acne Inversa (ICH), Department of Dermatology, Venereology and Allergology, Ruhr-University Bochum, Bochum, Germany; 2 Department of Dermatology, University Hospital Essen, Essen, Germany; 3 Department of Dermatology, Bielefeld University, Medical School and University Medical Center Ostwestfalen-Lippe, Klinikum Bielefeld Rosenhöhe, Bielefeld, Germany

**Keywords:** hidradenitis suppurativa, diet, mediterranean diet, meta-inflammation, metabolic syndrome, gut microbiome, obesity, diabetes

## Abstract

**Background:**

Dietary factors have been suggested to influence inflammatory skin diseases; however, their role in the pathogenesis and clinical course of hidradenitis suppurativa (HS) remains insufficiently understood. Increasing evidence suggests that HS is a systemic immunometabolic disease characterized by chronic low-grade inflammation and metabolic comorbidities such as obesity, insulin resistance, and metabolic syndrome. Dietary patterns may therefore influence HS activity through metabolic and inflammatory pathways.

**Objective:**

To systematically evaluate available evidence on dietary patterns, nutritional interventions, and micronutrient status in hidradenitis suppurativa and to assess their associations with disease onset, disease severity, and underlying metabolic and inflammatory mechanisms.

**Methods:**

A systematic search was conducted in PubMed/Medline for studies published between 1985 and 2026, following PRISMA guidelines. Eligible study types included observational studies, interventional trials, and case-control or cross-sectional studies investigating dietary exposures or nutritional interventions in HS. Reference lists were screened for additional records.

**Results:**

Eleven studies met the inclusion criteria. Across observational cohorts, lower adherence to Mediterranean-style dietary patterns, higher glycaemic dietary patterns, and micronutrient deficiencies, particularly vitamin D and zinc, were generally associated with greater HS disease severity. Interventional evidence was limited. A small pilot study reported clinical improvement following a very low-calorie ketogenic diet, and yeast-exclusion diets were associated with symptom improvement in small patient cohorts. Evidence from bariatric surgery cohorts suggested that malabsorptive procedures and persistent micronutrient deficiencies may be associated with worsening HS in some patients. Overall, the available studies suggest potential links between diet and HS through metabolic, inflammatory, and microbiome-related pathways, although the evidence remains limited and heterogeneous.

**Conclusion:**

Current evidence suggests that dietary habits and nutritional status may influence hidradenitis suppurativa through metabolic and inflammatory mechanisms. Although data remain heterogeneous and largely observational, promoting anti-inflammatory dietary patterns, optimizing micronutrient intake, and addressing obesity may offer supportive benefits alongside standard HS therapies. Further controlled studies are needed to establish causality.

## 1 Introduction

Hidradenitis suppurativa (HS) is a chronic inflammatory disease of the intertriginous skin characterized by follicular occlusion, rupture of the pilosebaceous unit, and subsequent inflammation (1, 2). It affects approximately 1–4% of the population, typically arises after puberty, and is more common in women (3, 4). Clinically, HS presents with recurrent painful nodules, abscesses, and sinus tract formation, frequently resulting in scarring and substantial impairment in quality of life (1).

Beyond mechanical follicular occlusion, HS is increasingly recognized as a complex immune-mediated disorder associated with systemic comorbidities. Activation of innate and adaptive immune pathways, including inflammasome signaling and pro-inflammatory cytokines such as TNF-α, IL-1β, and IL-17, contributes to sustained disease activity, chronic inflammation, and tissue destruction (2).

Obesity, smoking, and metabolic syndrome are common comorbidities. In particular, obesity shows strong associations with disease severity, potentially through mechanical friction as well as obesity-related systemic inflammation (5–9). These observations support the emerging concept of HS as a systemic immunometabolic disease characterized by chronic low-grade metainflammation, linking metabolic dysfunction with persistent immune activation. Current European guidelines recommend severity stratification using Hurley staging and the IHS4 score and propose individualized treatment strategies including topical therapies, systemic antibiotics, hormonal agents, retinoids, biologics, and surgery (10, 11). Although newer biologics such as secukinumab and bimekizumab have expanded therapeutic options, many patients continue to experience incomplete responses or recurrent disease (12–14).

Given the strong association between HS and metabolic comorbidities, modifiable lifestyle factors have gained increasing interest. Dietary factors have been extensively investigated in inflammatory dermatoses such as acne vulgaris, psoriasis, and atopic dermatitis, where nutritional patterns have been shown to influence metabolic and inflammatory signaling pathways. However, their role in HS remains less clearly defined.

Emerging evidence suggests that high-glycaemic and dairy-rich diets may exacerbate inflammatory activity via insulin-resistance and androgen-mediated pathways, while observational data and case reports describe potential improvement with dairy restriction, low-glycaemic diets, or brewer’s-yeast avoidance (15–17). Conversely, anti-inflammatory dietary patterns, particularly the Mediterranean diet (MD), have been proposed as potential modulators of systemic inflammation (17–20). Nevertheless, available evidence remains heterogeneous and largely based on retrospective analyses and self-reported outcomes.

Several reviews have previously discussed dietary factors in HS, however, many were published several years ago and do not reflect recent advances in the understanding of HS as a systemic immunometabolic disease. In particular, emerging evidence linking dietary patterns with metabolic dysfunction, gut microbiome alterations, and chronic low-grade inflammation has not been comprehensively integrated in the context of HS. Therefore, this systematic review aims to synthesize the most recent evidence on dietary patterns, nutritional interventions, and micronutrient status in HS and to evaluate their potential associations with disease onset, severity, and underlying metabolic and inflammatory pathways. By integrating current findings from nutritional, metabolic, and microbiome research, this review seeks to clarify the potential role of diet as a modifiable factor in HS and to inform future research as well as supportive lifestyle-based management strategies.

## 2 Methods

A systematic literature search was conducted in PubMed/MEDLINE in April 2025 to identify studies investigating the relationship between dietary factors, metabolic alterations, and HS. The reference lists of relevant articles were additionally screened to identify further eligible studies that may not have been captured by the database search. Prior to manuscript submission, the database search was repeated in March 2026 to capture newly published literature.

The review was conducted in accordance with the Preferred Reporting Items for Systematic Reviews and Meta-Analyses (PRISMA) guidelines. The research question was defined according to the PICOS framework as follows: population, patients diagnosed with hidradenitis suppurativa; exposure, dietary patterns, nutritional interventions, micronutrient status, or metabolic factors; comparator, healthy controls or alternative dietary exposures where applicable; outcomes, disease onset, disease severity, or metabolic and inflammatory markers associated with HS; and study design, observational or interventional human studies. A formal hierarchy of primary and secondary outcomes was not predefined due to the heterogeneity of the available literature.

Only articles published in English or German were considered eligible. Review articles, conference abstracts, letters to the editor, and studies not conducted in human populations were excluded. Eligible study designs included observational and interventional studies such as cohort studies, case–control studies, cross-sectional studies, retrospective chart reviews, and pilot or interventional trials investigating dietary exposures or nutritional factors in patients with HS.

**Figure 1:**
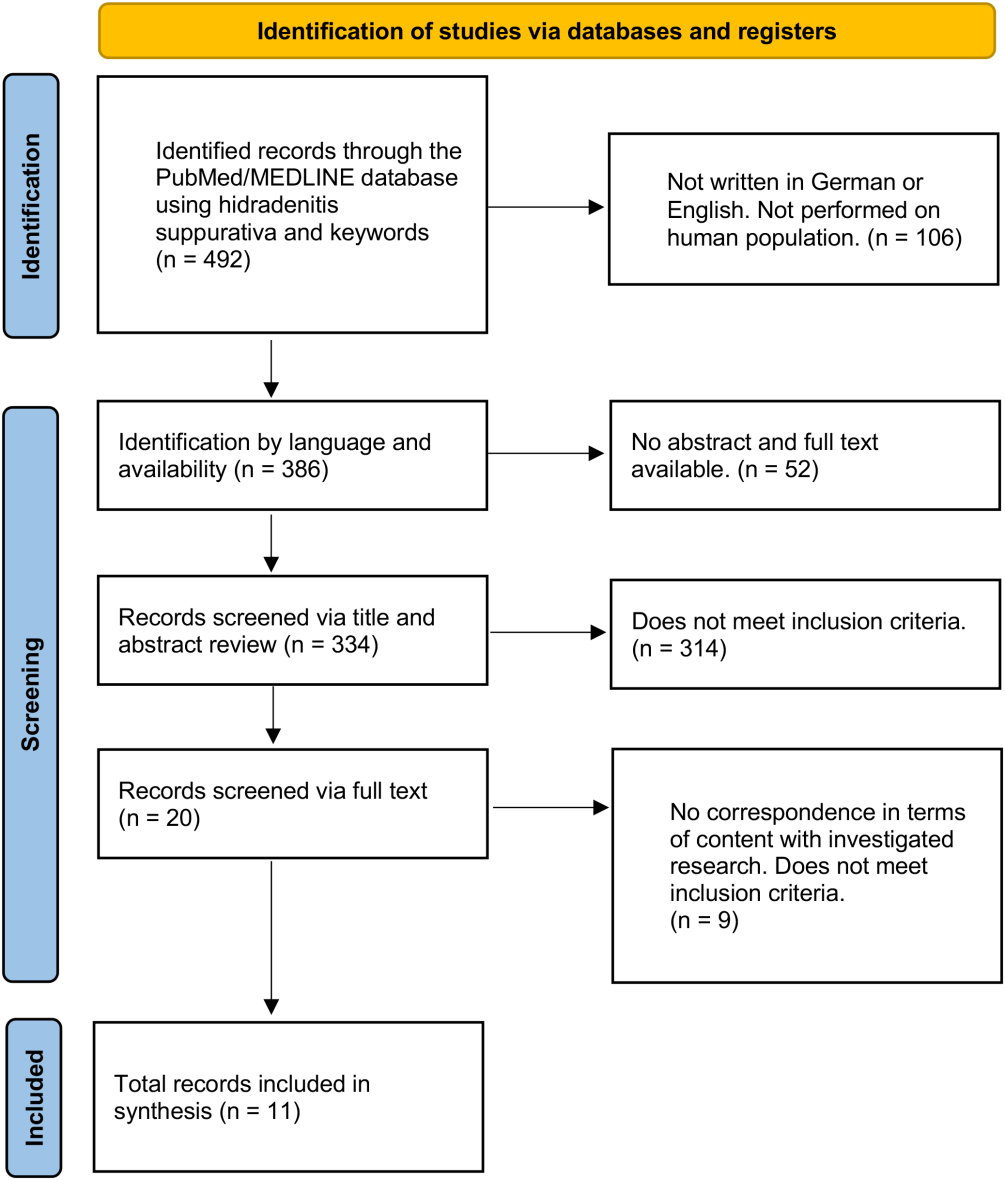
PRISMA flow diagram of study selection process. The diagram illustrates the identification, screening, eligibility, and inclusion of studies in this systematic review. A total of 492 records were identified through database searching. After removal based on language and availability criteria, 334 records were screened by title and abstract. Of these, 314 records were excluded. Twenty full-text articles were assessed for eligibility, of which 9 were excluded due to not meeting inclusion criteria. Ultimately, 11 studies were included in the qualitative synthesis. Source: Page MJ, et al. BMJ 2021;372:n71. doi: 10.1136/bmj.n71. This work is licensed under CC BY 4.0. To view a copy of this license, visit https://creativecommons.org/licenses/by/4.0/.

Titles and abstracts were initially screened for relevance. Full texts of potentially eligible studies were subsequently assessed for eligibility according to the predefined inclusion criteria. Screening and study selection were performed independently by two reviewers. Disagreements regarding study inclusion were resolved through discussion and consultation with a third reviewer. The study selection process is illustrated in **Figure 1**.

The search strategy combined terms related to hidradenitis suppurativa with terms describing dietary factors, nutritional components, and metabolic parameters. The search was primarily based on predefined free-text terms. To enhance sensitivity and account for variations in indexing, selected Medical Subject Headings (MeSH) terms were incorporated to complement the free-text search. The following search terms were used: (“hidradenitis suppurativa” OR “acne inversa”) AND (“fish” OR “milk” OR “vegetables” OR “sugar” OR “meat” OR “tomato” OR “potato” OR “eggplant” OR “grains” OR “omega-3” OR “carbohydrates” OR “gluten” OR “wheat” OR “nutrition” OR “obesity” OR “vitamin” OR “salt” OR “fats” OR “zinc” OR “Mediterranean diet” OR “caffeine” OR “alcohol” OR “cheese” OR “dairy products” OR “pork” OR “beef” OR “glycemic index” OR “rice” OR “beer” OR “yeast” OR “olive oil” OR “legumes” OR “turmeric” OR “ginger”).

For each included study, relevant information was extracted including study design, sample size, patient characteristics, dietary or metabolic exposure, clinical outcomes related to HS severity, and key findings, which are summarized in **Table 1**. These data were summarized qualitatively in order to identify recurring patterns and areas of agreement or heterogeneity across studies. Data were synthesized narratively without formal stratified analyses, and no *a priori* subgrouping was defined.

**Table 1:** Summary of included studies on dietary exposures and interventions in hidradenitis suppurativa (HS).

First author (Year)	Country	Study design	N (HS/control)	Exposure/intervention	Key findings	Risk of bias
Cannistrà (2014) (17)	Italy	Observational follow-up	12/—	Brewer’s yeast–exclusion diet (diet + surgery)	Sustained remission with yeast exclusion; relapse upon reintroduction	Moderate
Garcovich (2019) (21)	Italy	Retrospective, cohort	12/—	Bariatric surgery (malabsorptive vs restrictive)	*De novo* or aggravated HS after malabsorptive procedures; persistent micronutrient deficiencies	High
Barrea (2018) (18)	Italy	Cross-sectional	41/41	MD adherence (PREDIMED), body composition (PhA)	Lower MD adherence and PhA; both inversely correlated with disease severity	Low
Barrea (2021) (22)	Italy	Case-control cross-sectional	35/35	TMAO levels and dietary intake	Higher TMAO correlated with greater HS severity; supports diet–microbiome–inflammation link	Moderate
Velluzzi (2021) (23)	Italy	Case–control	35/35	MD adherence, physical activity, anthropometry	HS patients had higher adiposity and lower health scores; no link between diet or activity and severity; severity correlated with disease duration	Moderate
Lorite-Fuentes (2022) (19)	Spain	Cross-sectional	221/—	MD adherence and physical activity	Higher MD adherence and physical activity associated with lower HS severity; extra-virgin olive oil use linked to reduced disease activity	Low
Seetan (2022) (24)	Jordan	Comparative cross-sectional	110/110	Serum vitamin D levels	HS patients were vitamin D deficient; no correlation with disease severity	Moderate
Bouwman (2024)(20)	Netherlands	Population-based cohort	1004/5000	Diet quality and physical activity	Poorer diet scores and less physical activity associated with higher HS likelihood and severity	Moderate
Kesik (2024) (15)	Turkey	Cross-sectional case–control	50/50	Dietary pattern analysis: MD adherence (MEDAS), glycaemic index, nutrient intake, body composition	Lower MD adherence and higher glycaemic index associated with greater HS severity; MEDAS inversely correlated with IHS4	Moderate
Verde (2024) (25)	Italy	Pilot interventional study	12/—	VLCKD	VLCKD reduced HS activity and improved metabolic/inflammatory biomarkers	Low
Lelonek (2024) (26)	Poland	Cross-sectional	40/40	Gut microbiome composition and dietary habits	HS associated with gut dysbiosis and distinct dietary profiles; supports gut–skin axis role	Moderate

HS, hidradenitis suppurativa; MD, Mediterranean diet; MEDAS, Mediterranean Diet Adherence Screener; PREDIMED, Prevención con Dieta Mediterránea score; PhA, phase angle; TMAO, trimethylamine N-oxide; VLCKD, very low-calorie ketogenic diet.

Risk of bias was assessed qualitatively by considering study design, sample size, and potential methodological limitations, including selection bias, recall bias, and confounding factors such as obesity, smoking status, and comorbid metabolic disease.

## 3 Results

### 3.1 Dietary patterns and disease severity

Recent observational studies have evaluated dietary patterns, nutritional status, and lifestyle behaviors in HS, with particular attention to the MD adherence and physical activity (15, 18–20, 23). Across these cohorts, a consistent nutritional profile emerges: HS patients exhibit substantially lower MD adherence, higher consumption of processed and high-glycaemic foods, and reduced intake of nutrient-dense foods such as fruits, whole grains, legumes, nuts, fish, and unsweetened dairy (15, 19, 20).

In the case-control study by Kesik et al. (15), HS patients demonstrated a higher dietary glycaemic index, lower vitamin C and iron intake, and markedly poorer MD adherence (mean MEDAS 4.3 vs. 6.0; 86% classified as low adherers). Several MD-related components, including use of extra-virgin olive oil as the primary culinary fat, intake of >4 tablespoons of olive oil per day, and ≥3 weekly servings of nuts, were associated with lower disease severity. Disease severity correlated positively with glycaemic index, total energy and fat intake, BMI, waist circumference, and consumption of refined sugar and highly processed foods, while MEDAS score, physical activity, and vitamin C and zinc intake showed inverse correlations. In multivariate analyses, MD adherence remained the only independent predictor of HS severity.

Lorite-Fuentes et al. (19) evaluated adherence to the MD and its association with disease severity in 221 Spanish patients: higher MD adherence and specific MD components (notably extra-virgin olive oil use and preference for poultry over red meat) were independently associated with milder disease. Physical activity positively correlated with MD adherence and showed a trend toward lower severity.

In the largest dataset to date, Bouwman et al. (20) conducted a nested case-control study within the longitudinal Lifelines Cohort Study in the Northern Netherlands, including 1004 adult HS patients and 5000 age-matched controls. Dietary intake was assessed using a validated food frequency questionnaire and translated into the Lifelines Diet Score (LLDS), alternate Mediterranean Diet Score (aMED), and Dutch Dietary Guidelines score (DDG). Compared to controls, HS patients consumed fewer calories, macronutrients, fruits, legumes, nuts, fish, whole grains, and unsweetened dairy products, while consuming more savory snacks, artificially sweetened products, and white meat. LLDS, aMED, and DDG scores were consistently lower in HS patients. Moreover, lower adherence to LLDS and DDG was associated with a higher likelihood of HS development, and physical activity levels were significantly reduced in the HS group.

Barrea et al. (18) further linked nutritional quality to cellular health: HS patients had significantly lower phase angle (PhA), a marker of cell membrane integrity and hydration, which strongly correlated with disease severity. Their dietary pattern was characterized by lower intake of complex carbohydrates, monounsaturated fatty acids (MUFA) and n-3 polyunsaturated fatty acids (PUFA), and higher intake of saturated fats and n-6 PUFA, resulting in an elevated n-6/n-3 ratio and increased ox-LDL levels, indicative of a pro-inflammatory metabolic state.

In a matched case-control study from Sardinia (35 HS patients and 35 controls), Velluzzi et al. (23) assessed MD adherence using the Med Diet Score (MDS) and disease severity using the Hurley stage system and the Sartorius score. HS patients had lower MDS values and less favorable anthropometric indicators than controls; however, no significant correlations were observed between MD adherence and disease severity. Instead, the Sartorius score correlated positively with HS duration, which the authors attributed to diagnostic delay.

### 3.2 Targeted dietary interventions

Beyond observational associations, few studies have investigated structured dietary interventions in HS. Verde et al. (25) evaluated a very low-calorie ketogenic diet (VLCKD) in twelve women with HS and obesity. The 28-day ketogenic phase induced nutritional ketosis through severe caloric and carbohydrate restriction using standardized meal replacements. Patients experienced a 24% reduction in Sartorius score, accompanied by significant decreases in BMI, waist circumference, and fat mass. PhA increased markedly, aligning with earlier findings that PhA, reflecting cellular integrity and intracellular hydration, is reduced in HS and improves alongside reductions in inflammation.

Improvements were also observed in metabolic and oxidative stress markers. Circulating levels of trimethylamine N-oxide (TMAO), oxidized low-density lipoprotein (oxLDL), and derivatives of reactive oxygen metabolites (dROMs) decreased significantly during the intervention period. Changes in these biomarkers were positively correlated with changes in the Sartorius score. In addition, a significant improvement in dermatology-related quality of life, measured by the DLQI, was reported.

Evidence also indicates that brewer’s yeast may exacerbate HS symptoms. Brewer’s yeast is a unicellular fungus widely used in fermentation processes and present in various foods including baked products, fermented beverages, and certain cheeses. It naturally occurs in items like bread, pastries, beer, wine, soy-based sauces, vinegar, and mold-ripened cheeses. The potential involvement of this microorganism as a trigger or aggravating factor in the pathogenesis of HS remains a matter of ongoing discussion. Cannistrà et al. (17) examined twelve patients with recurrent axillary and perineal fistulas who underwent surgical management combined with a brewer’s yeast-free diet. All patients showed a specific IgG-mediated immune response to brewer’s yeast and wheat and were placed on a strictly controlled yeast-free diet for 12 months with monthly follow-up. Stabilization of clinical symptoms and gradual regression of skin lesions were observed. Recurrence of lesions occurred after reintroduction of yeast-containing foods, such as beer and baked goods. Patients also reported improvements in quality of life and daily activities, including sexual activity in those with inguinal or perigenital involvement.

A retrospective descriptive study of a hospital-based HS cohort (n = 178) identified 12 patients with prior bariatric surgery (BS). In ten cases, HS developed *de novo* following predominantly malabsorptive procedures, with a median latency of two years. Post-BS patients frequently presented with moderate to severe disease and persistent micronutritional deficiencies (75%), affecting zinc, vitamin D, vitamin A, vitamin B12, and iron levels. Baseline zinc and vitamin D levels were available for 62 HS patients without prior BS from the same cohort. Post-BS cases demonstrated significantly lower median serum zinc levels (42.5 vs. 83.0 μg/L; p = 0.005), whereas vitamin D levels did not differ significantly. The authors discussed chronic malabsorption and persistent suppuration as well as potential contributing factors including disturbance of the gut microbiome, adipokine dysregulation, extracellular matrix changes, and increased skin friction after weight loss (21).

### 3.3 Micronutrient status and deficiencies

Results from bariatric cohorts have indicated that micronutrient deficiencies, particularly low levels of zinc, vitamin D, vitamin A, vitamin B12, and iron, may influence HS activity. Further insights into micronutrient intake arise from the cohort previously reported by Kesik et al. (15), which also identified lower levels of several nutrients in HS patients compared with controls. Specifically, reduced consumption of vitamin C and iron was observed, with vitamin C intake showing inverse associations with both IHS4 scores and Hurley stage, while zinc intake correlated negatively with Hurley stage only. The biological plausibility for these associations is supported by the role of vitamin C in collagen stabilization, ceramide synthesis, wound healing, and antioxidant defense mechanisms relevant to inflammatory skin conditions such as HS.

A comparative cross-sectional study from Jordan further evaluated serum vitamin D levels in 110 HS patients and 110 matched healthy controls. All patients with HS were vitamin D deficient, with mean concentrations of 8.4 ng/mL compared to 17.6 ng/mL in controls. None of the patients had sufficient levels. Smoking and elevated BMI were significantly more common among HS cases than controls. Regression analyses identified smoking and obesity as independent predictors of lower vitamin D levels. However, no significant correlation was observed between vitamin D concentrations and HS disease severity (24).

### 3.4 Gut microbiome and metabolic byproducts

Several studies have investigated the gut microbiome and related metabolic products in HS, given their close relationship with dietary habits and nutrient intake. In a study by Lelonek et al. (26), gut microbiome composition in HS patients and healthy controls was analyzed using next-generation sequencing. Overall microbial diversity, assessed by alpha-diversity and Shannon diversity indices, did not differ significantly between groups. However, compositional analyses revealed differences in the abundance of specific bacterial taxa, including the orders Desulfovibrionales, Clostridia, and Opitutales. Regression analyses demonstrated significant associations between BMI and the abundance of selected bacterial genera. In addition, coffee consumption was correlated with the presence of genera such as *Bilophila*, *Ruminococcus gauvreauii* group, and UCG-003. Dietary habits also differed between groups, with HS patients reporting higher sugar intake and more frequent milk consumption compared with controls.

In a case–control cross-sectional study, Barrea et al. (22) evaluated circulating TMAO levels in 35 treatment-naïve HS patients and 35 controls matched for sex, age, and BMI. HS patients showed significantly higher circulating TMAO levels, lower adherence to the MD, and lower PhA values compared with controls. Within the HS cohort, patients with Hurley stage II disease had higher BMI, waist circumference, total energy intake, and circulating TMAO levels, as well as lower PhA and lower adherence to the Mediterranean diet, compared with patients with Hurley stage I disease. Circulating TMAO levels correlated positively with Sartorius score, and this association remained significant after adjustment for BMI, waist circumference, MD adherence, total energy intake, and PhA. In linear regression analysis, circulating TMAO levels and PhA were identified as the main predictors of HS severity. In the VLCKD intervention study by Verde et al. (25), significant reductions in circulating TMAO were observed after 28 days, accompanied by decreases in dROMs and oxLDL and parallel improvements in Sartorius score. Correlation analyses in that study showed positive associations between changes in Sartorius score and changes in TMAO, dROMs, and oxLDL.

## 4 Discussion

In this systematic review, we synthesized evidence from eleven studies investigating the relationship between diet, metabolism, and inflammatory pathways in HS. Overall, the available evidence suggests that nutritional and metabolic factors may meaningfully influence disease activity. Across observational cohorts, higher adherence to Mediterranean-style dietary patterns and greater physical activity were generally associated with lower disease severity, whereas high-glycaemic diets, ultra-processed foods, and unfavorable body composition were consistently linked to worse clinical outcomes. Interventional evidence remains limited but provides preliminary support for the role of targeted metabolic dietary strategies. VLCKDs were associated with reductions in clinical severity scores, anthropometric measures, and biomarkers of oxidative stress, while yeast-exclusion diets demonstrated sustained remission in selected patient subgroups. In parallel, several studies identified micronutrient deficiencies, particularly zinc and vitamin D, as common findings in HS cohorts. Emerging data further indicate that microbiome-derived metabolites such as TMAO may reflect metabolic dysregulation and correlate with disease severity, highlighting the potential role of the gut-metabolism-inflammation axis in HS.

Taken together, these findings suggest that dietary influences in HS likely operate through broader metabolic and inflammatory pathways rather than isolated nutritional effects. Increasingly, HS is recognized as a systemic immunometabolic disease in which chronic cutaneous inflammation is closely linked to metabolic dysfunction. Epidemiological studies consistently report a higher prevalence of obesity, insulin resistance, metabolic syndrome, and type 2 diabetes mellitus among individuals with HS, indicating that the disease frequently develops within a systemic pro-inflammatory metabolic environment (27, 28). Chronic low-grade metainflammation is considered a central mechanism linking metabolic disturbances with inflammatory disease activity. In obesity, adipose tissue acts as an active endocrine and immunological organ characterized by macrophage infiltration and increased secretion of pro-inflammatory cytokines such as TNF-α, IL-6, and IL-1β, alongside reduced levels of anti-inflammatory adipokines including adiponectin. In parallel, hyperinsulinaemia may enhance insulin-like growth factor-1 signaling and activate mammalian target of rapamycin complex 1 (mTORC1)-dependent pathways that promote keratinocyte proliferation, follicular hyperkeratinization, and immune dysregulation, processes closely implicated in early HS pathogenesis (28, 29).

This pathophysiological framework is reflected in clinical observations linking obesity with HS prevalence and disease severity. Several anthropometric markers, including body mass index, body fat percentage, waist circumference, and waist-to-hip ratio, have been proposed as indicators associated with HS susceptibility and disease burden (30, 31). Beyond systemic inflammatory effects, obesity may also contribute to HS through mechanical mechanisms, as increased skin friction and occlusion in intertriginous regions can promote follicular rupture and lesion formation (28). Consistent with these observations, a high prevalence of overweight and obesity has been reported in HS cohorts, with some studies indicating that up to 84% of patients are classified as overweight or obese. Despite this high prevalence, referral to structured weight-management services appears to be limited in clinical practice, with reported rates as low as 12% among overweight or obese patients (32). A recent Mendelian randomization analysis demonstrated a significant positive association between genetically predicted body mass index and HS risk, supporting a potential causal link between obesity and disease development (31). These findings suggest that excess body weight may remain insufficiently addressed in routine clinical care and highlight the importance of improving recognition and management of obesity in HS populations.

Insulin resistance and type 2 diabetes mellitus provide an additional mechanistic bridge between metabolic dysfunction and HS pathogenesis. Clinical data indicate that diabetes mellitus is relatively common among patients with HS and is associated with increased disease severity, with a higher risk observed in individuals with more advanced disease stages, particularly those with Hurley stage III disease. Older age and higher BMI have been identified as additional independent risk factors (33). These observations are further supported by meta-analytic evidence demonstrating a substantially higher prevalence of diabetes in patients with HS compared with controls (34). Chronic inflammation itself may further aggravate insulin resistance, reinforcing a reciprocal interaction between metabolic and immune dysregulation.

Dietary composition may influence HS activity through its effects on metabolic and inflammatory signaling pathways. Observational evidence from studies included in this review supports a potential link between adherence to the MD and disease severity in HS, with higher adherence generally associated with lower disease activity (18, 19). The MD, characterized by high intake of vegetables, legumes, whole grains, fish, nuts, and extra-virgin olive oil, provides multiple anti-inflammatory substrates including monounsaturated fatty acids, omega-3 fatty acids, fiber, and polyphenols. These nutrients exert antioxidant effects, modulate gut microbiota composition, and promote the production of short-chain fatty acids, which influence immune regulation and intestinal barrier integrity (35, 36).

Nevertheless, heterogeneity across studies must be considered when interpreting these findings. Dietary intake was assessed using different indices, including MEDAS, PREDIMED, LLDS, and aMED, each capturing slightly different aspects of dietary quality and MD adherence. Variations in background dietary habits, BMI distribution, and concomitant pharmacological therapies may further contribute to differences across study populations. Beyond dietary adherence scores alone, markers of nutritional and cellular health such as PhA may better reflect the interaction between metabolic status and HS severity, as PhA explained greater variability in disease severity than MD adherence alone in one cohort (18).

Such variability may not only arise from methodological differences but also from broader contextual factors, including differences in habitual dietary patterns across populations. The available evidence included in this review is largely derived from European and West Asian cohorts, while data from East Asia, South Asia, North America, and South America remain limited. This represents an important limitation, particularly in dietary research, as nutritional patterns and lifestyle-related risk factors differ substantially across regions. Global epidemiological reviews have reported considerable heterogeneity in HS prevalence estimates and have highlighted the lack of representative prevalence data from many non-European regions (37, 38). Although regional differences in HS prevalence have been described, these findings should be interpreted cautiously because of heterogeneity in diagnostic methods, study design, healthcare access, coding practices, and population characteristics (37, 38).

Western dietary patterns, including those frequently described in North American and other highly industrialized populations, are often characterized by higher intake of refined carbohydrates, ultra-processed foods, animal-derived fats, saturated fats, and lower dietary fiber intake (39). In contrast, several traditional dietary patterns in Asian populations have been described as relatively richer in plant-derived foods, dietary fiber, vitamins, and antioxidants, although these patterns are highly heterogeneous and increasingly affected by urbanization and westernization (40). This dietary transition has been associated with alterations in gut microbial composition and increased metabolic disease risk in Asian populations, including insulin resistance, dyslipidaemia, and low-grade inflammation (40, 41). Although it is biologically plausible that such regional dietary differences may influence HS burden through effects on obesity, insulin resistance, gut microbiome composition, and systemic low-grade inflammation, these factors may partially contribute to the observed variation in HS prevalence across different geographic regions. However, direct evidence linking specific regional diets to HS prevalence is currently lacking. Therefore, geographic differences in HS prevalence should not be attributed to diet alone. Future studies should include more diverse populations and dietary patterns to clarify whether nutrition contributes to regional variation in HS epidemiology and disease severity.

Emerging evidence also highlights the potential role of the gut microbiome in the pathophysiology of HS. The gut microbiome interacts closely with dietary intake and metabolic processes and plays an important role in immune regulation. Although microbiome diversity itself does not appear to differ substantially between HS patients and controls in some studies, compositional shifts in specific microbial taxa have been reported (26). These alterations may influence microbial metabolic pathways and the production of microbiota-derived metabolites. One metabolite of particular interest is TMAO, which is generated from dietary precursors such as choline and carnitine through microbial metabolism and subsequent hepatic oxidation. Elevated circulating TMAO levels have been associated with systemic inflammation and cardiometabolic risk in several chronic diseases (42). In HS, higher circulating TMAO concentrations have been reported in association with greater disease severity and unfavorable metabolic profiles. Although these findings do not establish causality, they support the concept that microbiome-derived metabolites may represent a link between dietary habits, metabolic dysfunction, and inflammatory activity in HS.

In addition to dietary patterns, specific nutritional interventions have been explored as potential therapeutic strategies. Among these, ketogenic dietary approaches have gained interest due to their metabolic and anti-inflammatory effects. Preliminary interventional evidence from studies included in this review suggests that VLCKD may improve both clinical disease severity and metabolic parameters in patients with HS (25). These improvements appear to be accompanied by reductions in oxidative stress and metabolic dysfunction, supporting the role of metabolic pathways in disease activity.These effects may be mediated, at least in part, by improved insulin sensitivity and reduced hyperinsulinaemia, which could attenuate insulin- and IGF-1–driven mTORC1 signaling previously implicated in HS pathogenesis (43–45). In addition, ketone bodies such as β-hydroxybutyrate may exert direct anti-inflammatory effects through inhibition of the NLRP3 inflammasome (46). Given the close association between HS, insulin resistance, and type 2 diabetes mellitus, such metabolic improvements may contribute to reduced inflammatory disease activity. However, larger randomized longitudinal studies are needed to confirm the long-term efficacy and safety of this approach.

Additional evidence suggests that specific dietary triggers may contribute to disease exacerbation in susceptible individuals. Building on initial observations of symptomatic improvement with yeast-exclusion diets (17), Aboud et al. reported sustained clinical remission over six years in HS patients maintaining strict yeast avoidance, whereas reintroduction of yeast-containing foods consistently triggered relapses (47). Although based on small cohorts, these findings support the possibility that targeted dietary interventions may modulate inflammatory activity in selected HS subgroups.

Despite the potential benefits of dietary modification and moderate weight loss, evidence from bariatric cohorts suggests that rapid or malabsorptive weight reduction may aggravate HS in susceptible individuals. This effect may be mediated by nutritional deficiencies, particularly involving micronutrients such as zinc, vitamin D, vitamin A, vitamin B12, and iron, as well as alterations in metabolic and microbiome-related pathways (21). These findings underscore that iatrogenic malnutrition and microbiome perturbations may contribute to disease exacerbation. Excess skin after significant postoperative weight loss may also increase friction in intertriginous regions, aggravating lesions (48), while rapid tissue remodeling may trigger low-grade inflammation, collagen degradation, and elastic fiber loss, potentially sustaining disease activity (49).

Micronutrient deficiencies were frequently observed in HS, although their direct associations with severity remain inconsistent. Nevertheless, zinc and vitamin D play key immunomodulatory roles relevant to HS pathophysiology. Zinc regulates cytokine production, oxidative stress, and wound healing, and reduced serum levels are repeatedly reported in HS. Supplementation with 90 mg/day zinc gluconate, with or without nicotinamide, has shown reductions in flare frequency and disease severity in mild-to-moderate HS (50–53). Vitamin D similarly supports keratinocyte differentiation and immune regulation, with consistently lower serum levels documented in HS and small studies demonstrating clinical improvements following supplementation (52, 53).

This systemic metabolic-inflammatory interplay may also help explain the increased cardiovascular burden observed in patients with HS. Large cohort studies have demonstrated higher rates of major adverse cardiovascular events and increased cardiovascular mortality in individuals with HS compared with the general population. From a mechanistic perspective, this association appears plausible, as chronic systemic inflammation, obesity, insulin resistance, and metabolic syndrome are all established drivers of endothelial dysfunction and atherosclerotic disease (54). In HS, these factors frequently cluster and may therefore act synergistically to increase long-term cardiometabolic risk. In line with these findings, emerging proteomic evidence indicates widespread dysregulation of proteins related to cardiovascular and atherosclerotic pathways, as well as neutrophil-associated inflammatory responses (55).

The recognition of HS as a systemic immunometabolic disease has therefore prompted increasing interest in therapeutic strategies that target metabolic pathways, which may open new avenues for therapeutic intervention. In particular, glucagon-like peptide-1 receptor agonists (GLP-1 RAs), such as liraglutide and semaglutide, represent a biologically plausible therapeutic approach. These agents improve glycaemic control, enhance insulin sensitivity, and promote weight reduction through central appetite regulation in the hypothalamus, while also exerting anti-inflammatory effects (56). Preliminary clinical observations suggest that GLP-1 RAs may also influence HS disease activity (57). In small observational studies of HS patients with obesity, treatment with liraglutide or semaglutide was associated with significant weight reduction and improvements in quality of life, with some studies also reporting reductions in inflammatory markers and HS severity scores (57–59). These findings suggest that the potential benefits of GLP-1 RAs in HS may arise from their combined effects on obesity-related inflammation and systemic immune activation. Although current evidence remains limited, these observations further support the concept of HS as an immunometabolic disease and highlight GLP-1 receptor agonists as a promising adjunctive therapeutic strategy, particularly in patients with obesity-associated HS phenotypes.

Although treatment aspects were not the primary focus of this review, the present findings may have important implications for the therapeutic conceptualization of HS. Current treatment strategies are largely guided by severity-based classifications such as the Hurley staging system and IHS4 score (10), which primarily reflect the extent of cutaneous disease. However, the evidence synthesized in this review suggests that HS activity is closely linked to systemic metabolic dysfunction and chronic low-grade inflammation.

In this context, integrating metabolic parameters into the therapeutic framework of HS may represent a complementary approach to existing severity-based models. Factors such as obesity, insulin resistance, and metabolic syndrome could be considered not only as comorbidities but as modulators of disease activity and potential therapeutic targets. This perspective supports a more stratified management approach, in which patients with metabolically driven disease phenotypes may benefit from adjunctive interventions targeting metabolic pathways, including dietary modification, weight management, and metabolically active pharmacological agents. While current evidence remains insufficient to redefine treatment algorithms, these findings highlight the potential value of expanding the therapeutic definition of HS beyond purely lesion-based criteria toward a more integrated immunometabolic model of disease management.

## 5 Limitations

Several limitations should be considered when interpreting the findings of this systematic review. First, important limitations arise from the methodological characteristics of the included studies. Most studies were observational in nature, including cross-sectional, retrospective, or case–control designs with relatively small sample sizes, limiting statistical power and the ability to infer causal relationships. Dietary exposure was assessed using heterogeneous instruments, which complicates direct comparison across studies and may introduce measurement variability. Similarly, HS severity was evaluated using different clinical indices such as the IHS4, Hurley staging, and Sartorius score, further contributing to methodological heterogeneity. In addition, the level of standardization of dietary interventions was limited. Nutritional strategies ranged from observational assessments of dietary adherence to small interventional approaches, often without standardized protocols or consistent duration of intervention. Reliance on self-reported dietary intake and lifestyle behaviors may introduce recall and reporting bias. Moreover, important confounders, such as smoking status, BMI, metabolic comorbidities, and concurrent systemic or biologic therapies, were inconsistently controlled across studies. Several cohorts included patients receiving antibiotics or biologic treatments, which may independently influence inflammatory activity, metabolic parameters, and dietary behaviors, thereby potentially confounding observed associations.

Second, this systematic review itself has several methodological limitations. The literature search was restricted to PubMed/MEDLINE and to studies published in English and German, which may have introduced both database and language bias and led to the exclusion of relevant studies indexed in other databases such as Embase or Scopus or published in other languages. In addition, although a comprehensive search strategy and manual reference screening were applied, the possibility remains that relevant studies were missed due to differences in indexing terms, unpublished data, or grey literature not captured in the search process. Furthermore, the search strategy relied in part on predefined Boolean combinations of food- and nutrient-related terms, which may have limited the identification of studies using broader or alternative terminology.Publication bias must therefore also be considered, as studies reporting negative or null associations may be underrepresented in the available literature. These limitations highlight the need for more rigorous research in this field. Future studies should prioritize well-designed prospective cohort studies and randomized controlled trials with standardized dietary interventions, validated dietary assessment tools, and uniform HS severity measures. Larger multicenter investigations that integrate metabolic, immunological, and microbiome-related biomarkers may further help to clarify causal relationships between nutrition, metabolic dysfunction, and HS activity and support the development of evidence-based dietary recommendations for clinical practice.

## 6 Conclusion

Emerging evidence suggests that dietary factors may influence HS activity through interconnected metabolic and inflammatory pathways. The recognition of HS as a systemic immunometabolic disease highlights the importance of metabolic comorbidities such as obesity, insulin resistance, and type 2 diabetes mellitus, which may contribute to inflammatory disease activity. In this context, dietary modification and metabolically targeted therapies may represent promising adjunctive strategies within HS management.

From a clinical perspective, these findings support the integration of nutritional assessment and counseling into a multimodal treatment approach for HS. Rather than adopting universal dietary recommendations, future care models should emphasize individualized strategies that consider metabolic comorbidities, body composition, disease severity, and lifestyle factors. Incorporating structured nutritional counseling into dermatologic care pathways may represent a feasible and low-risk strategy to complement pharmacologic and surgical treatments while addressing the systemic metabolic burden associated with HS. Future research should prioritize well-designed prospective studies and randomized controlled trials to clarify causal relationships between dietary factors and HS activity, identify patient subgroups most likely to benefit from nutritional interventions, and establish evidence-based dietary recommendations. Integrating metabolic and nutritional strategies into comprehensive HS care may ultimately improve both dermatologic outcomes and long-term cardiometabolic health in affected patients.

## Data Availability

The original contributions presented in the study are included in the article/supplementary material. Further inquiries can be directed to the corresponding authors.

## References

[1] Ballard K Sathe NC Shuman VL . Hidradenitis suppurativa. In: StatPearls. StatPearls Publishing, Treasure Island (FL (2026). 30521288

[2] Sabat R Jemec GBE Matusiak Ł Kimball AB Prens E Wolk K . Hidradenitis suppurativa. Nat Rev Dis Primers. (2020) 6:18. doi: 10.1038/s41572-020-0149-1. PMID: 32165620

[3] Margesson LJ Danby FW . Hidradenitis suppurativa. Best Pract Res Clin Obstet Gynaecol. (2014) 28:1013–27. doi: 10.1016/j.bpobgyn.2014.07.012. PMID: 25214437

[4] van der Weijden DAY Koerts NDK van Munster BC van der Zee HH Horváth B . Hidradenitis suppurativa tarda: defining an understudied elderly population. Br J Dermatol. (2023) 190:105–13. doi: 10.1093/bjd/ljad317. PMID: 37665963

[5] Patel ZS Hoffman LK Buse DC Grinberg AS Afifi L Cohen SR . Pain, psychological comorbidities, disability, and impaired quality of life in hidradenitis suppurativa. Curr Pain Headache Rep. (2017) 21:49. doi: 10.1007/s11916-017-0647-3. PMID: 29094219 PMC5784845

[6] Khan A Chang MW . The role of nutrition in acne vulgaris and hidradenitis suppurativa. Clin Dermatol. (2022) 40:114–21. doi: 10.1016/j.clindermatol.2022.04.001. PMID: 35398509

[7] Krajewski PK Matusiak Ł Szepietowski JC . Adipokines as an important link between hidradenitis suppurativa and obesity: a narrative review. Br J Dermatol. (2023) 188:320–7. doi: 10.1093/bjd/ljac107. PMID: 36641766

[8] Agnese ER Tariche N Sharma A Gulati R . The pathogenesis and treatment of hidradenitis suppurativa. Cureus. (2023) 15:e49390. doi: 10.7759/cureus.49390. PMID: 38146560 PMC10749691

[9] Canard C Diaz Cives A Gaubil-Kaladjian I Bertin E Viguier M . Impact of bariatric surgery on hidradenitis suppurativa. Acta Derm Venereol. (2021) 101:adv00471. doi: 10.2340/00015555-3830. PMID: 34003299 PMC9380277

[10] Zouboulis CC Bechara FG Benhadou F Bettoli V Bukvić Mokos Z Del Marmol V . European S2k guidelines for hidradenitis suppurativa/acne inversa part 2: treatment. J Eur Acad Dermatol Venereol. (2025) 39:899–941. doi: 10.1111/jdv.20472. PMID: 39699926 PMC12023723

[11] Ocker L Abu Rached N Seifert C Scheel C Bechara FG . Current medical and surgical treatment of hidradenitis suppurativa: a comprehensive review. J Clin Med. (2022) 11:7240. doi: 10.3390/jcm11237240. PMID: 36498816 PMC9737445

[12] Martora F Megna M Battista T Potestio L Annunziata MC Marasca C . Adalimumab, ustekinumab, and secukinumab in the management of hidradenitis suppurativa: a review of the real-life experience. Clin Cosmet Investig Dermatol. (2023) 16:135–48. doi: 10.2147/CCID.S391356. PMID: 36698446 PMC9869696

[13] Sonnet M . Welches Biologikum wirkt bei HS am besten? hautnah Dermatol. (2025) 41:23. doi: 10.1007/s15012-025-8815-3. PMID: 30311153

[14] Calabrese L Cartocci A Rubegni P French LE Kendziora B . Efficacy and safety of biologics for hidradenitis suppurativa: a network meta-analysis of phase III trials. J Eur Acad Dermatol Venereol. (2026) 40:637–45. doi: 10.1111/jdv.20617. PMID: 40062409 PMC13014433

[15] Kesik F Dogan-Gunaydin S Fisunoglu M . The impact of diet on hidradenitis suppurativa severity: a cross-sectional case-control study. Med (Kaunas). (2024) 60:2107. doi: 10.3390/medicina60122107. PMID: 39768986 PMC11678350

[16] Shen AS Johnson JS Kerns ML . Dietary factors and hidradenitis suppurativa. Dermatol Ther (Heidelb). (2023) 13:3007–17. doi: 10.1007/s13555-023-01056-1. PMID: 37899421 PMC10689602

[17] Cannistrà C Finocchi V Trivisonno A Tambasco D . New perspectives in the treatment of hidradenitis suppurativa: surgery and brewer’s yeast-exclusion diet. Surgery. (2013) 154:1126–30. doi: 10.1016/j.surg.2013.04.018. PMID: 23891479

[18] Barrea L Fabbrocini G Annunziata G Muscogiuri G Donnarumma M Marasca C . Role of nutrition and adherence to the Mediterranean diet in the multidisciplinary approach of hidradenitis suppurativa: evaluation of nutritional status and its association with severity of disease. Nutrients. (2018) 11:57. doi: 10.3390/nu11010057. PMID: 30597889 PMC6356593

[19] Lorite-Fuentes I Montero-Vilchez T Arias-Santiago S Molina-Leyva A . Potential benefits of the Mediterranean diet and physical activity in patients with hidradenitis suppurativa: a cross-sectional study in a Spanish population. Nutrients. (2022) 14:551. doi: 10.3390/nu14030551. PMID: 35276909 PMC8840522

[20] Bouwman K Moazzen S Kroah-Hartman M Dijkstra G Horváth B Alizadeh BZ . Diet and physical activity as risk-reducing factors for hidradenitis suppurativa. J Eur Acad Dermatol Venereol. (2024) 38:910–9. doi: 10.1111/jdv.19726. PMID: 38116943

[21] Garcovich S De Simone C Giovanardi G Robustelli E Marzano AV Peris K . Post-bariatric surgery hidradenitis suppurativa: a new patient subset associated with malabsorption and micronutritional deficiencies. Clin Exp Dermatol. (2019) 44:283–9. doi: 10.1111/ced.13732. PMID: 30144136

[22] Barrea L Muscogiuri G Pugliese G de Alteriis G Maisto M Donnarumma M . Association of trimethylamine N-oxide with the clinical severity of hidradenitis suppurativa. Nutrients. (2021) 13:1997. doi: 10.3390/nu13061997. PMID: 34200594 PMC8226830

[23] Velluzzi F Anedda J Pisanu S Dell’Antonia M Deledda A Boi A . Mediterranean diet, lifestyle and quality of life in Sardinian patients affected with hidradenitis suppurativa. J Public Health Res. (2021) 11:2706. doi: 10.4081/jphr.2021.2706. PMID: 34850622 PMC8958440

[24] Seetan K Eldos B Saraireh M Omari R Rubbai Y Jayyusi A . Prevalence of low vitamin D levels in patients with hidradenitis suppurativa in Jordan: a comparative cross-sectional study. PloS One. (2022) 17:e0265672. doi: 10.1371/journal.pone.0265672. PMID: 35303020 PMC8932615

[25] Verde L Cacciapuoti S Caiazzo G Megna M Martora F Cavaliere A . Very low-calorie ketogenic diet in the management of hidradenitis suppurativa: an effective and safe tool for improvement of the clinical severity of disease. Results of a pilot study. J Transl Med. (2024) 22:149. doi: 10.1186/s12967-024-04853-0. PMID: 38350939 PMC10863195

[26] Lelonek E Szepietowski JC . Insights into gut microbiome composition in hidradenitis suppurativa: a comprehensive examination of dietary habits and environmental influences. Nutrients. (2024) 16:1776. doi: 10.3390/nu16111776. PMID: 38892709 PMC11174550

[27] Mintoff D Benhadou F Pace NP Frew JW . Metabolic syndrome and hidradenitis suppurativa: epidemiological, molecular, and therapeutic aspects. Int J Dermatol. (2022) 61:1175–86. doi: 10.1111/ijd.15910. PMID: 34530487

[28] Mintoff D Agius R Benhadou F Das A Frew JW Pace NP . Obesity and hidradenitis suppurativa: targeting meta-inflammation for therapeutic gain. Clin Exp Dermatol. (2023) 48:984–90. doi: 10.1093/ced/llad182. PMID: 37171791

[29] Abu Rached N Gambichler T Dietrich JW Ocker L Seifert C Stockfleth E . The role of hormones in hidradenitis suppurativa: a systematic review. Int J Mol Sci. (2022) 23:15250. doi: 10.3390/ijms232315250. PMID: 36499573 PMC9736970

[30] Vossen ARJV van der Zee HH Onderdijk AJ Boer J Prens EP . Hidradenitis suppurativa is not associated with the metabolic syndrome based on body type: a cross-sectional study. J Dermatol. (2017) 44:154–9. doi: 10.1111/1346-8138.13572. PMID: 27608328

[31] Kjærsgaard Andersen R Riis PT Zachariae C Thomsen SF Quin L Dinh KM . Hidradenitis suppurativa and smoking, obesity, psoriasis, inflammatory bowel disease, and systemic sclerosis: results from a two-sample Mendelian randomization study. JAMA Dermatol. (2026) 162:142–50. doi: 10.1001/jamadermatol.2025.5010. PMID: 41405899 PMC12712827

[32] Khalil N Hussain K Patel NP . Low referral rate of overweight/obese patients with hidradenitis suppurativa to weight-management services: a missed opportunity? Clin Exp Dermatol. (2024) 49:383–5. doi: 10.1093/ced/llad425. PMID: 38037674

[33] Abu Rached N Gambichler T Ocker L Dietrich JW Quast DR Sieger C . Screening for diabetes mellitus in patients with hidradenitis suppurativa: a monocentric study in Germany. Int J Mol Sci. (2023) 24:6596. doi: 10.3390/ijms24076596. PMID: 37047569 PMC10094965

[34] Bui TL Silva-Hirschberg C Torres J Armstrong AW . Hidradenitis suppurativa and diabetes mellitus: a systematic review and meta-analysis. J Am Acad Dermatol. (2018) 78:395–402. doi: 10.1016/j.jaad.2017.08.042. PMID: 29056237

[35] Estruch R Ros E Salas-Salvadó J Covas MI Corella D Arós F . Primary prevention of cardiovascular disease with a Mediterranean diet supplemented with extra-virgin olive oil or nuts. N Engl J Med. (2018) 378:e34. doi: 10.1056/NEJMoa1800389. PMID: 29897866

[36] De Filippis F Pellegrini N Vannini L Jeffery IB La Storia A Laghi L . High-level adherence to a Mediterranean diet beneficially impacts the gut microbiota and associated metabolome. Gut. (2016) 65:1812–21. doi: 10.1136/gutjnl-2015-309957. PMID: 26416813

[37] Jfri A Nassim D O’Brien E Gulliver W Nikolakis G Zouboulis CC . Prevalence of hidradenitis suppurativa: a systematic review and meta-regression analysis. JAMA Dermatol. (2021) 157:924–31. doi: 10.1001/jamadermatol.2021.1677. PMID: 34037678 PMC8156162

[38] Phan K Charlton O Smith SD . Global prevalence of hidradenitis suppurativa and geographical variation: a systematic review and meta-analysis. BioMed Dermatol. (2020) 4:2. doi: 10.1186/s41702-019-0052-0. PMID: 38164791

[39] Cordain L Eaton SB Sebastian A Mann N Lindeberg S Watkins BA . Origins and evolution of the Western diet: health implications for the 21st century. Am J Clin Nutr. (2005) 81:341–54. doi: 10.1093/ajcn.81.2.341. PMID: 15699220

[40] Therdtatha P Shinoda A Nakayama J . Crisis of the Asian gut: associations among diet, microbiota, and metabolic diseases. Biosci Microbiota Food Health. (2022) 41:83–93. doi: 10.12938/bmfh.2021-085. PMID: 35854695 PMC9246424

[41] Misra A Khurana L Isharwal S Bhardwaj S . South Asian diets and insulin resistance. Br J Nutr. (2008) 101:465–73. doi: 10.1017/S0007114508073649. PMID: 18842159

[42] Caradonna E Abate F Schiano E Paparella F Ferrara F Vanoli E . Trimethylamine-N-oxide (TMAO) as a rising-star metabolite: implications for human health. Metabolites. (2025) 15:220. doi: 10.3390/metabo15040220. PMID: 40278349 PMC12029716

[43] Maarouf M Platto JF Shi VY . The role of nutrition in inflammatory pilosebaceous disorders: implication of the skin-gut axis. Australas J Dermatol. (2019) 60:e90–8. doi: 10.1111/ajd.12909. PMID: 30175843

[44] Melnik BC . Linking diet to acne metabolomics, inflammation, and comedogenesis: an update. Clin Cosmet Investig Dermatol. (2015) 8:371–88. doi: 10.2147/CCID.S69135. PMID: 26203267 PMC4507494

[45] Melnik B . Dietary intervention in acne: attenuation of increased mTORC1 signaling promoted by Western diet. Dermatoendocrinol. (2012) 4:20–32. doi: 10.4161/derm.19828. PMID: 22870349 PMC3408989

[46] Youm YH Nguyen KY Grant RW Goldberg EL Bodogai M Kim D . The ketone metabolite beta-hydroxybutyrate blocks NLRP3 inflammasome-mediated inflammatory disease. Nat Med. (2015) 21:263–9. doi: 10.1038/nm.3804. PMID: 25686106 PMC4352123

[47] Aboud C Zamaria N Cannistrà C . Treatment of hidradenitis suppurativa: surgery and yeast-exclusion diet. Results after 6 years. Surgery. (2020) 167:1012–5. doi: 10.1016/j.surg.2019.12.015. PMID: 32098690

[48] Kromann CB Ibler KS Kristiansen VB Jemec GB . The influence of body weight on the prevalence and severity of hidradenitis suppurativa. Acta Derm Venereol. (2014) 94:553–7. doi: 10.2340/00015555-1800. PMID: 24577555

[49] Musale V Wasserman DH Kang L . Extracellular matrix remodelling in obesity and metabolic disorders. Life Metab. (2023) 2:load021. doi: 10.1093/lifemeta/load021. PMID: 37383542 PMC10299575

[50] Hessam S Sand M Meier NM Gambichler T Scholl L Bechara FG . Combination of oral zinc gluconate and topical triclosan: an anti-inflammatory treatment modality for initial hidradenitis suppurativa. J Dermatol Sci. (2016) 84:197–202. doi: 10.1016/j.jdermsci.2016.08.010. PMID: 27554338

[51] Brocard A Knol AC Khammari A Dréno B . Hidradenitis suppurativa and zinc: a new therapeutic approach. A pilot study. Dermatology. (2007) 214:325–7. doi: 10.1159/000100883. PMID: 17460404

[52] Fabbrocini G Marasca C Luciano MA Guarino M Poggi S Fontanella G . Vitamin D deficiency and hidradenitis suppurativa: the impact on clinical severity and therapeutic responsivity. J Dermatolog Treat. (2021) 32:843–4. doi: 10.1080/09546634.2020.1714538. PMID: 31994944

[53] Guillet A Brocard A Bach Ngohou K Graveline N Leloup AG Ali D . Verneuil’s disease, innate immunity and vitamin D: a pilot study. J Eur Acad Dermatol Venereol. (2015) 29:1347–53. doi: 10.1111/jdv.12857. PMID: 25512084

[54] Egeberg A Gislason GH Hansen PR . Risk of major adverse cardiovascular events and all-cause mortality in patients with hidradenitis suppurativa. JAMA Dermatol. (2016) 152:429–34. doi: 10.1001/jamadermatol.2015.6264. PMID: 26885728

[55] Glickman JW David E Shokrian N Hawkins K Duca ED Hu BD . Large-scale blood proteomic analysis across different inflammatory skin conditions reveals extensive immune dysregulation with distinct biomarker profiles. Allergy. (2026) 81:524–38. doi: 10.1111/all.70157. PMID: 41246813

[56] Karacabeyli D Lacaille D . Glucagon-like peptide 1 receptor agonists in patients with inflammatory arthritis or psoriasis: a scoping review. J Clin Rheumatol. (2024) 30:26–31. doi: 10.1097/RHU.0000000000001949. PMID: 36870080

[57] Krajewski PK Złotowska A Szepietowski JC . The therapeutic potential of GLP-1 receptor agonists in the management of hidradenitis suppurativa: a systematic review of anti-inflammatory and metabolic effects. J Clin Med. (2024) 13:6292. doi: 10.3390/jcm13216292. PMID: 39518431 PMC11547001

[58] Nicolau J Nadal A Sanchís P Pujol A Masmiquel L Nadal C . Liraglutide for the treatment of obesity among patients with hidradenitis suppurativa. Med Clin (Barc). (2024) 162:118–22. doi: 10.1016/j.medcli.2023.11.007. PMID: 38044187

[59] Alkhalili S Hailemichael R Allen C Martin SA Blaszczak A Flood KS . A single-center retrospective study of GLP-1 agonist use in patients with hidradenitis suppurativa. Arch Dermatol Res. (2025) 317:940. doi: 10.1007/s00403-025-04443-0. PMID: 30311153

